# Nonoperative treatment of slipped capital femoral epiphysis: a scientific study

**DOI:** 10.1186/1749-799X-6-10

**Published:** 2011-02-19

**Authors:** Pedro Carlos MS Pinheiro

**Affiliations:** 1 Post-Graduation Departament of the Federal University of Rio de Janeiro, (UFRJ) and Jesus Children's Hospital, Rio de Janeiro, Brazil

## Abstract

**Background:**

Treatment of the Slipped Capital Femoral Epiphysis remains a cause of concern due to the fact that the true knowledge of the etiopathogeny is unknown, as well as one of its major complications: *chondrolysis*. The conservative treatment remains controversial; it has been overlooked in the studies and subjected to intense criticism. The purpose of this study is to investigate the results of treatment on the hip of patients displaying slipped capital femoral epiphysis, using the plaster cast immobilization method and its link to chondrolysis.

**Methods:**

The research was performed based on the study of the following variables: symptomatology, and the degree of slipping. A hip *spica *cast and bilateral short/long leg casts in abduction, internal rotation with anti-rotational bars were used for immobilizing the patient's hip for twelve weeks. Statistical analysis was accomplished by Wilcoxon's marked position test and by the Fisher accuracy test at a 5% level.

**Results:**

A satisfactory result was obtained in the acute group, 70.5%; 94%; in the chronic group (chronic + acute on chronic). Regarding the degree of the slipping, a satisfactory result was obtained in 90.5% of hips tested with a mild slip; in 76% with moderate slip and 73% in the severe slip. The statistical result revealed that a significant improvement was found for flexion (p = 0.0001), abduction (p = 0.0001), internal rotation (p = 0.0001) and external rotation (p = 0.02). Chondrolysis was present in 11.3% of the hips tested. One case of pseudoarthrosis with aseptic capital necrosis was presented. There was no significant variation between age and chondrolysis (p = 1.00).Significant variation between gender/non-white patients versus chondrolysis (p = 0.031) and (p = 0.037), respectively was verified.

No causal association between plaster cast and chondrolysis was observed (p = 0.60). In regard to the symptomatology group and the slip degree *versus *chondrolysis, the p value was not statistically significant in both analyses, p = 0.61 and p = 0.085 respectively.

**Conclusions:**

After analyzing the nonoperative treatment of slipped capital femoral epiphysis and chondrolysis, we conclude that employment of the treatment revealed that the method was functional, efficient, valid, and reproducible; it also can be used as an alternative therapeutic procedure regarding to this specific disease.

## Background

The contributions and reasons for the use of the non-operative management of Slipped Capital Femoral Epiphysis (SCFE) are as follows:

- ***applicability***: non-operative treatment of SCFE allows the use of this method at any hospital, even for surgeons who have very little hands-on experience with this specific disease;

- ***elucidation***: the work elucidates the employment of a principle and the method of treatment little exploited by world literature;

- ***knowledge***: this research offers the opportunity for orthopedic surgeons to employ a method based on biology, contributing to further knowledge of SCFE, thereby also promoting the possibility of a wide debate on the subject;

- ***reproducible***: the easy use of this method allows the treatment to be repeated in other innovating medicine centers by an execution of a general procedure to a widespread application, adding value to knowledge;

- ***results***: the work has proven its effectiveness based on statistical data obtained, thereby demonstrating its importance and feasibility;

- ***therapeutic*: **the use of the plaster cast method revealed the possibility of obtaining favorable results for its use;

- ***prognosis***: early diagnosis, associated with the simplicity of the SCFE method, favors a good prognosis and low morbidity for the disease.

This work posits that the benefits and application of the therapeutic criteria based on biology comprise a valid method of treatment, considering disease prognostic uncertainty.

## Patients and Methods

The Committee of Ethics of the Jesus Children's Hospital in Brazil, Rio de Janeiro, have analyzed and approved the Research Project entitled, Nonoperative treatment of slipped capital femoral epiphysis, which was also evaluated by the Ethics Committee for Research of the Federal University of Rio de Janeiro (UFRJ), Brazil.

The typology of the design employed in this sample was a study of a single cohort with observational, longitudinal and retrospective characteristics. In this research, chondrolysis was the dependent variable. A consecutive series of 106 hip joints in eighty-four patients affected, the great majority of them obese, displaying SCFE, were treated by means of plaster cast (Table 1 and Table 2). Patients' age varied at the time of diagnosis, ranging from 7.6 to 15.8 years. The duration of the follow-up ranged from 12 months, with the complete growth-plate closure, to 146 months, an average of 51 months. Thirteen patients were younger than eleven, 55 patients were between the ages of 11 and 13; and 16 patients were between the ages of 13 and 16. The average age was 12.5, males having the average age of 14.5 and females 10.5. Forty-four patients were males, and 40 females. Regarding race, 43 were white, and 41 were non-white. Unilateral involvement was present in 62 left hips and 44 right hips. Bilateral displacement (simultaneous involvement) of hips was present in 19 patients. Three patients were detected as displaying involvement of the contra lateral hip in different periods (sequential bilaterality), comprising, in total, 22 bilateral slip patients.

**Table 1:** Data on the Patients

Case	Age at Diagnosis *(Yrs.)*	Sex*	Race^#^	Hip Treated^¥^	Classification	Grade of Slip	Type of Traction	Time in cast (*Days*)	Type of cast	Follow-up Analysis *(Months)*
1	11.8	F	N-W	R	Chronic	Mild	Skin	198	1 1/2 *Spica*	144

2	11.6	F	N-W	L	Chronic	Mild	-	106	1 1/2 *Spica*	96

3	10.6	M	W	L	Chronic	Mild	-	93	1 1/2 *Spica*	66

F	11		W	R+L	Acute, Acute	Severe, Severe	Skin Skin	84	Double S*pica*	116 116

5	12.1	F	N-W	L	Acute	Moderate	Skin	114	Double Long Leg Casts	60

6	12.6	F	N-W	L	Chronic	Mild	Skin	119	1 1/2 *Spica*	144

7	9.10 11.3	F	W	R+L	Acute, Acute	Mild, Mild	Skin, Skin	84, 84	1 1/2 *Spica*, 1 1/2 *Spica*	126 108

8	13	M	N-W	L	Chronic	Severe	Skeletal	90	1 1/2 *Spica*	50

9	12.2	F	W	R	Chronic	Mild	Skin	119	1 1/2 *Spica*	60

10	11.4	F	N-W	R	Chronic	Mild	Skin	119	1 1/2 *Spica*	57

11	12	F	N-W	L	Acute	Mild	Skin	91	1 1/2 *Spica*	52

12	11	F	N-W	R	Chronic	Moderate	Skin	77	1 1/2 *Spica*	84

13	11.7	F	N-W	R	Acute	Moderate	Skin	84	1 1/2 *Spica*	146

14	12.8	M	W	R	Chronic	Mild	Skin	105	1 1/2 *Spica*	12

15	13.10	M	N-W	L	Chronic	Mild	Skin	84	1 1/2 *Spica*	118

16	10.2	F	N-W	R+L	Acute, Chronic	Mild, Mild	_ _	91	Double *Spica*	45 45

17	12	M	N-W	R	Chronic	Moderate	-	91	1 1/2 *Spica*	58

18	11.9	F	N-W	R+L	Chronic, Chronic	Mild, Moderate	Skin, Skin	84	Double *Spica*	12 12

19	14	M	N-W	L	Chronic	Moderate	Skin	101	1 1/2 *Spica*	43

20	12.2	F	N-W	R	Chronic	Mild	-	84	1 1/2 *Spica*	65

21	12.6	M	W	R+L	Chronic, Chronic	Moderate, Moderate	Skin, Skin	84	Double *Spica*	48 48

22	8.3	M	W	L	Chronic	Mild	Skin	119	1 1/2 *Spica*	32

23	10.8	F	NW	L	Chronic	Mild	Skin	84	1 1/2 *Spica*	76

24	12.1	F	W	R+L	Chronic, Chronic	Mild, Moderate	Skin, Skin	98	Double S*pica*	41 41

25	9	F	N-W	R	Chronic	Mild	Skin	84	1 1/2 *Spica*	75

26	12.5	F	W	L	Acute on Chronic	Mild	Skin	84	1 1/2 *Spica*	23

27	12.8	M	N-W	R+L	Acute, Acute	Mild, Moderate	Skin, Skin	84	Double S*pica*	71 71

28	11.10	F	N-W	R	Acute	Mild	Skin	84	1 1/2 *Spica*	70

29	13.5	M	N-W	R+L	Chronic, Chronic	Mild, Moderate	Skin, Skin	84	Double S*pica*	28 28

30	11.5	F	N-W	R+L	Chronic, Chronic	Mild, Mild	Skin, Skin	84	Double S*pica*	78 78

31	14	M	W	R	Chronic	Mild	-	84	1 1/2 *Spica*	35

32	11.6	F	W	L	Chronic	Mild	-	84	1 1/2 *Spica*	25

33	10.8	F	W	L	Chronic	Moderate	-	84	1 1/2 *Spica*	68

34	14	M	W	L	Chronic	Mild	-	84	1 1/2 *Spica*	122

35	11.8	M	W	L	Acute on Chronic	Severe	-	80	1 1/2 *Spica*	56

36	12	F	N-W	R	Chronic	Mild	-	81	1 1/2 *Spica*	130

37	9.7	F	N-W	R	Chronic	Mild	-	80	1 1/2 *Spica*	48

38	11.8	M	W	L	Acute	Mild	-	88	1 1/2 *Spica*	48

39	12.1	M	N-W	R+L	Chronic, Chronic	Mild, Mild	-	88 88	1 1/2*Spica*, Bilateral Short Casts	46 12

40	13	M	W	R	Chronic	Mild	-	83	1 1/2 *Spica*	50

41	11.9	F	W	R+L	Chronic, Chronic	Mild, Mild	- _	85	Double *spica*	81 81

42	14.5	M	W	L	Chronic	Mild	-	84	1 1/2 *Spica*	13

43	11.9	M	N-W	L	Chronic	Mild	-	84	1 1/2 *Spica*	45

*M = male and F = female; # W = white and N-W = non-white; ¥ R = right and L = left

**Table 2:** Data on the Patients

Case	Age at Diagnosis *(Yrs.)*	Sex*	Race^#^	Hip Treated^¥^	Classification	Grade of Slip	Type of Traction	Time in cast (*Days*)	Type of cast	Follow-up Analysis *(Months)*
44	13	M	W	R+L	Chronic, Chronic	Severe, Mild	Skeletal, Skin	84	Double S*pica*	12 12

45	12	M	W	L	Chronic	Mild	-	84	1 1/2 *Spica*	45

46	11.7	F	N-W	L	Chronic	Severe	-	88	1 1/2 *Spica*	44

47	11.4	M	W	L	Chronic	Mild	-	84	1 1/2 *Spica*	48

48	12.6	F	N-W	R	Chronic	Mild	-	90	1 1/2 *Spica*	30

49	12.2	M	W	R+L	Chronic, Chronic	Severe, Mild	Skeletal _	93 90	1 1/2*Spica*, Bilateral Short Casts	50 36

50	11.8	M	W	L	Chronic	Mild	-	84	1 1/2 *Spica*	36

51	11.3	F	W	R+L	Chronic, Chronic	Mild, Mild	-	84	Double S*pica*	34 34

52	12	F	N-W	R	Chronic	Mild	-	84	1 1/2 *Spica*	36

53	12	F	W	R+L	Chronic, Chronic	Mild, Moderate	- _	95	Double *Spica*	12 12

54	14.3	M	N-W	L	Chronic	Mild	-	84	1 1/2 *Spica*	16

55	11	M	N-W	L	Chronic	Mild	-	88	1 1/2 *Spica*	64

56	11.3	F	W	L	Chronic	Moderate	-	89	1 1/2 *Spica*	50

57	12	F	W	R	Acute on Chronic	Mild	-	84	1 1/2 *Spica*	37

58	7.6	M	N-W	R	Acute	Mild	-	87	Bilateral Short Casts	94

59	12.9	M	N-W	R+L	Chronic, Chronic	Mild, Mild	-	82	Double S*pica*	19 19

60	11.8	F	W	L	Acute	Mild	-	87	Bilateral Short Casts	18

61	11.8	M	N-W	L	Chronic	Mild	-	106	Bilateral Short Casts	52

62	11.7	M	W	R+L	Chronic, Chronic	Mild, Severe	-	94	Bilateral Short Casts, Bilateral Long Leg Casts	13 13

63	13	M	N-W	R	Chronic	Mild	-	94	Bilateral Short Casts	13

64	11.2	F	W	R	Chronic	Mild	-	84	Bilateral Short Casts	48

65	11.4	M	W	L	Chronic	Mild	-	87	Bilateral Short Casts	50

66	13	M	N-W	L	Acute on Chronic	Severe	-	92	Bilateral Short Casts	43

67	9.9	F	N-W	R+L	Chronic, Chronic	Mild, Mild	-	90 90	Bilateral Short Casts	12

68	11.10	F	N-W	R	Chronic	Moderate	-	87	Bilateral Short Casts	12

69	13.6	M	W	L	Chronic	Mild	-	90	Bilateral Short Casts	36

70	10.5	M	W	R	Acute	Mild	-	90	Bilateral Short Casts	72

71	12.6	M	W	R	Chronic	Moderate	-	90	Bilateral Short Casts	12

72	12.1	F	W	R+L	Chronic, Chronic	Mild, Mild	-	93 93	Bilateral Short Casts	74

73	11.4	M	W	R+L	Chronic, Chronic	Mild, Mild	-	91	Bilateral Short Casts	70

74	11.5	M	W	L	Chronic	Mild	**-**	97 93	Bilateral Short Casts	58

75	12.10	F	N-W	L	Acute	Severe	Skeletal	90	Bilateral Short Casts	45

76	12.8	F	W	L	Chronic	Moderate	-	100	Bilateral Short Casts	38

77	15.8	M	W	L	Chronic	Moderate	-	90	Bilateral Short Casts	54

78	11.8	M	W	R+L	Chronic, Chronic	Mild, Moderate	-	90 90	Bilateral Short Casts	20

79	13.7	M	N-W	L	Chronic	Severe	Skeletal	107	Bilateral Short Casts	42

80	15.6	M	W	L	Chronic	Moderate	-	90	Bilateral Short Casts	12

81	12.8	F	N-W	L	Chronic	Mild	-	101	Bilateral Short Casts	37

82	13.9	M	W	R+L	Chronic, Chronic	Moderate, Mild	-	90 90	Bilateral Short Casts	14

83	12.7	M	W	L	Acute	Mild	-	90	Bilateral Short Casts	25

84	14	M	N-W	L	Chronic	Mild	-	97	Bilateral Short Casts	33

85	14	F	W	No	Chronic	Severe	-	-	-	48

86	11.8	F	N-W	No	Chronic	Mild	-	-	-	72

*M = male and F = female;

#W = white and N-W = non-white;

¥ R = right and L = left.

The methods used were evaluated based on symptomatology, and categorized as acute, chronic, or acute on chronic, according to Fahey and O'Brien [1]; also, slip degrees were documented by the standard method of thirds and classified as mild, moderate, or severe, according to Wilson, Jacobs, Schecter [2]; MacEwen and Ramsey who use the three grades of slip percentage [3]. The hips were systematically evaluated roentgenographically, as well as functionally, according to Heyman and Herndon's criteria [4], being also categorized as satisfactory and unsatisfactory by means of Aadalen, Weiner, Hoyt, Herdon and Herdon's criteria [5]. The radiographic methods used to analyze joint cartilage and detect chondrolysis were based on Ingram, Clarke, Clark and Marshall's criteria [6].

### Treatment Protocol

The main objective of the SCFE treatment is to avoid progressive displacement, with the use of the safest and the most effective technique to arrest growth plate. The routine methodology employed was based on the conservative principle with the use of *spicas *(earlier cases) and bilateral short/long leg casts in abduction, and a slight internal rotation (15°) with antirotational bars (later cases), aiming at immobilizing the patient's hip for 12 weeks.

Skin traction was used in order to avoid slip progression pre-casting in those patients displaying muscle spasms. Traction was also used to limit the patient's motion in order to reduce pain, and to prevent irritability (pain when moved through passive or active range of motion) [7]. Skeletal traction was also applied. This type of traction was used in these patients in an attempt to improve the neck-femoral head relationship. Reduction of the degree of slip by skeletal traction was not found in this series. For this reason, this type of traction was abandoned in SCFE pre-treatment.

Anaesthesia was administered as needed in the presence of pain and/or discomfort during plaster hip *spica *and short/long-leg cast application, in preparation for resting the hip.

Manipulation under anesthesia was performed as an alternative procedure to improve epiphysis position. In very few cases, Leadbetter's maneuver was gently applied prior to cast application, with the intention of improving the displacement of the neck/femoral head relationship, this being carefully carried out in chosen hips [8].

Cast immobilization was carried out for 12 weeks, in accordance with the casting protocol. No weightbearing was permitted during the "casting period". A hip *spica *was used in earlier cases; as time went on, and we gained more "experience" in the matter, choice was made of changing the method of plastering to short leg casts, on account of this being an easier application, allowing the patients to set hips and knees into motion in flexion and extension, thus performing muscle exercises (dynamic method). This type of immobilization was based on King's work, being also used to facilitate the patient's movement in a wheelchair [9].

**Figure 1:**
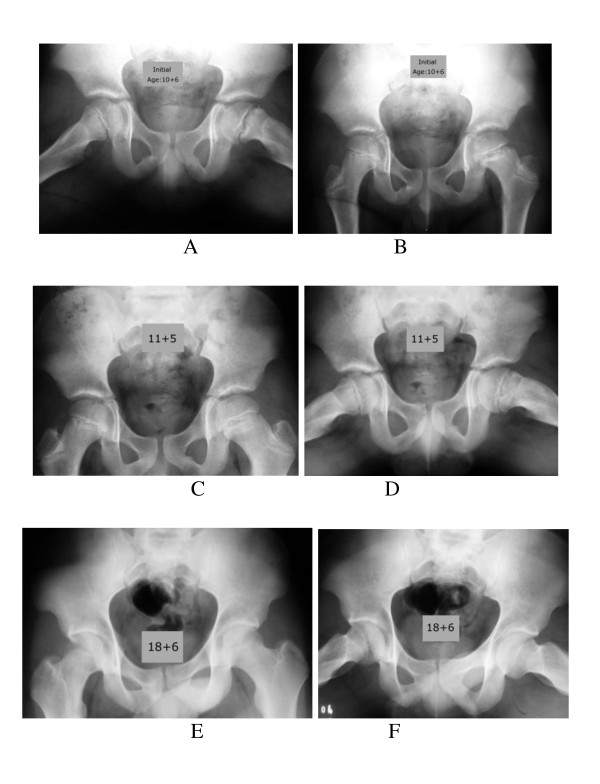
**Early slipping of the femoral epiphysis of the left hip**. A cast after twelve weeks was applied. The image of the left hip shows growth arrest andno progression with conservative management. (A and B) Anteroposterior and frog-leg lateral radiographs of the pelvis made before treatment, showing the zone of rarefaction on the metaphyseal side in the left hip of the growth plate in Chronic/Mild SCFE, in a ten and half year old boy. (C and D) Anteroposterior and frog-leg lateral radiographs eight months after spica cast had been discontinued. The rarefaction zone has diminished and persists in the left hip. (E and F) Final result. The growth-plate has completely closed on both radiographs of the left hip.

The criteria adopted for interruption of the plaster cast use were based on the physeal stability of the head with the femoral neck in the affected hip. Stability, which is the ability to walk without hip pain, was reached regardless of the progress and stage of the growth-plate closure (12 weeks). Follow-up was performed every three months to monitor the growth plate closure (Figure 1).

For patients who developed chondrolysis, the treatment protocol for the hip was as follows: analgesics, skin traction, bed rest, gentle active range-of-motion exercises, hydrotherapeutic/physiotherapeutic program, and the use of crutches (prolonged and nonweightbearing). The patients who presented chondrolysis underwent an observation period which took from 3 (three) to 12 (twelve) months; the criterion to stop the treatment for chondrolysis was opted for when irreversible clinical range of motion and deformation of both the femoral head and acetabulum were detected.

## Results

The results of the *spica *treatment (69%) and bilateral short/long leg casts (31%) in abduction and internal rotation with anti-rotational bars were evaluated functionally as well as roentgenographically according to Heyman, Herdon [4], Aadalen, Weiner, Hoyt, Herdon and Herdon's methods and criteria [5]. A 70.5% satisfactory result was obtained in the acute group, 94% in the chronic group (chronic + acute-on-chronic). Regarding the degree of the slipping, a satisfactory result was obtained in 90.5% of hips with a mild slip, 76% of hips with a moderate slip and 73% of hips with a severe slip.

It became necessary to reapply a new cast (re-displacement), after the established protocol (12 weeks), in six (5.6%) patients (Cases 25, 27, 63, 64, 74, and 75), who presented a second slip (average: 11 months after cast was discontinued) (Table 3).

**Table 3:** Distribution of the results of the six patients who presented a re-displacement (Progression cases after cast discontinued)

Cases	Age	Sex	Race	Hip	Physis Stage	Type of cast	Time in Cast
25	10+07	Female	Non-White	Right	Open	1 1/2 *Spica*	84

27	13+11	Male	Non-White	Right	Open	Double *Spica*	84

63	12+01	Female	White	Right	Open	Short Leg Casts	84

64	12+01	Female	White	Right	Open	Short Leg Casts	84

74	13+04	Male	White	Left	Open	Short Leg Casts	97

75	13+06	Female	Non-White	Left	Open	Long Leg Casts	90

**Figure 2:**
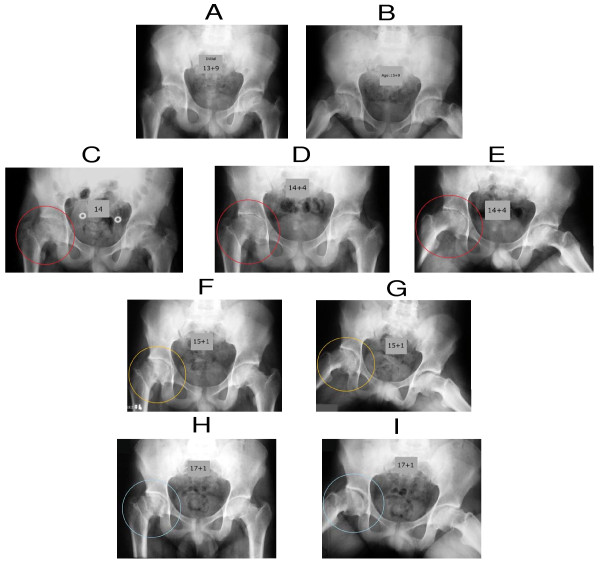
**Necrosis of the joint cartilage (Waldenström disease) of the right hip after cast period**. The functional  value of mobility of the affected hip was reached. Reversible clinical range of motion and deformation of both the femoral head and acetabulum were detected. (A and B)-Anteroposterior and frog-leg lateral radiographs of the pelvis made before treatment, showing bilateral chronic SCFE, being moderate slip in the right hip and mild in the left. (C) Anteroposterior roentgenogram of both hips after cast treatment with bilateral leg casts in abduction and an internal rotation. We may observe narrowing and irregularity of the right hip joint with demineralization of the surrounding bone = chondrolysis of the right hip. (D and E) Anteroposterior and frog-leg lateral radiographs of the hips showing closure of the growth-plate in the right hip, further demineralization with obliteration of the joint space and irregularity of the head of the femur and acetabulum and also decrease in cartilage thickness. (F and G) Anteroposterior and frog-leg lateral radiographs observing in the right hip some restoration of cartilage, with irregular contour of the femoral head. (H and I) Anteroposterior and frog-leg lateral radiographs observing in the right hip joint, the articular space is now widened compared to the initials X-rays. The femoral head presents mild deformity and limited range-of-motion in the right hip.

In 106 analyzed hips, 12 (11.3%) were detected with chondrolysis, clinically diagnosed by pain, limp, muscle spasms, stiffness, mobility limitations and narrowing of the hip joints' space, as radiographically determined. Among 44 males, only two (Cases 54 and 82) presented chondrolysis, and, in 40 females, eight (Cases 1,2,5,6,13,18,53 and 67) also displayed the same problem (Table 4). Among twelve hips with chondrolysis, four (33% [Cases 2, 5, 6, and 82]) presented transient chondrolysis, joints had widened close to normal, osteopenia had improved and pain and stiffness had decreased during the follow-up period (Figure 2).

**Table 4:** Chondrolysis incidence correlated to the following variables: sex, race, side, cast type, symptomatology and slip degree

Cases	Sex	Race	Hips	Cast Type	Symptomatology	Slip Degree
1	Female	Non-White	Right	1 1/2 *Spica*	Chronic	Mild

02	Female	Non-White	Left	1 1/2 *Spica*	Chronic	Mild

05	Female	Non-White	Left	Long Leg Casts	Acute	Moderate

06	Female	Non-White	Left	1 1/2 *Spica*	Chronic	Mild

13	Female	Non-White	Right	1 1/2 *Spica*	Acute	Moderate

18 Both	Female	Non-White	Right +Left	Double *Spica*	Chronic	Mild

53	Female	White	Left	Double *Spica*	Chronic	Moderate

54	Male	Non-White	Left	1 1/2 *Spica*	Chronic	Mild

67 Both	Female	Non-White	Right + Left	Short Leg Casts	Chronic	Mild

82	Male	White	Right	Short Leg Casts	Chronic	Moderate

Regarding race types, there were 43 white SCFE patients. Only two (Cases 54 and 82) displayed chondrolysis. Among 41 non-white patients, eight (Cases 1, 2, 5, 6, 13, 18, 54 and 67) also presented chondrolysis. Seven of these (Cases 1, 2, 5, 6, 13, 18, and 67) were female patients, and one was a male (Case 54).

In 19 patients (38 hips) with simultaneous involvement displacement, only two patient cases, 18 and 67, developed complications. In 44 hips with the right side affected, only three (Cases 1, 13 and 82) presented chondrolysis; in 62 cases on the left side, five (Cases 2, 5, 6, 53 and 54) presented the same complication.

Regarding the type of plaster cast used and chondrolysis, the following was observed: 1 1/2 *spica *- four chondrolysis hips, cases, (1, 2, 13 and 54); double short leg casts-three chondrolysis hips, cases (67 [both hips] and 82); double *spica *- three chondrolysis hips (18 [both hips] and 53); and double long leg casts-one chondrolysis hip (Case 5).

There were 17 hips with symptoms classified as acute, two (Cases 5 and 13), displaying chondrolysis, only ten hips (Cases 1, 2, 6, 18 [both hips], 54, 67 [both hips], 53 and 82) from 85 pertaining to the chronic group developed chondrolysis.

**Figure 3:**
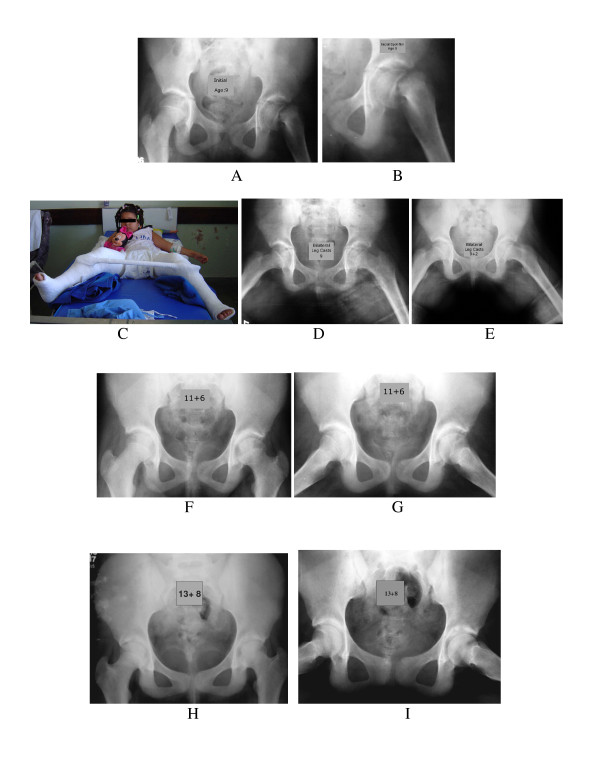
**Young female patient with severe slip of the left hip, treated by immobilization (anti-rotation plasters) after hip manipulation**. The range of motion of the left hip was normal at the final follow-up. (A and B) Anteroposterior radiograph of the pelvis and spot film before treatment, in a nine-year-old girl who had an acute/severe slip SCFE in the left hip. (C and D) Patient under general anesthesia submitted to gentle Leadbetter manipulation. Bilateral toe-to-groin casts had been applied. (E and F) Anteroposterior and Frog-leg lateral radiographs showing the physis beginning the closure process in AP and lateral views. (G and H) Anteroposterior and frog-leg lateral radiographs of the left hip, showing complete closure of the growth-plate.

Seventy-four displacements were observed in the mild-degree group. Seven hips (Cases 1, 2, 6, 18, 54, and 67 [both hips]) presented chondrolysis; in the moderate degree, 5 out of 21 hips (Cases 5, 13, 18, 53 and 82) presented chondrolysis, and none of the nine hips with a severe degree developed it. Avascular necrosis was not detected in none of the hips manipulated, by the Leadbetter maneuver [8] (Figure 3). Two patients with SCFE (Cases 85 and 86) were excluded from the study as these had the epiphyseal line already closured in the first appointment. Both patients had chondrolysis without any previous kind of treatment.

One case of pseudoarthrosis (0.9%) with necrosis of the head was detected after a repeated slip. This complication was classified as severe, of the traumatic displacement type, in the patient's hip (Case 75), due to a prolonged heavy femoral and tibia skeletal traction time employed simultaneously; avascular necrosis also was observed as a complication.

### Statistical Analysis

One of the objectives of the statistical analysis was to specify whether a significant variation existed in hip mobility measures (in degrees) before or after treatment. The absolute variation (in degrees) between pre-and post-treatment is given by the following formula: Absolute variation of flexion = flexion in post-treatment-flexion in pre-treatment. Statistical analysis was accomplished by Wilcoxon's marked positions test [10]. According to hip flexion analysis, significant variations (p = 0.0001) were found, i. e., there was an increase of 29.5° on average after treatment. With regard to hip abduction, a significant variation (p = 0.0001) was found, i. e., there was an increase of 12.5°. As for hip internal rotation, there were significant variations (p = 0.0001), i. e., an increase of 11.8°. Concerning hip external rotation, significant variations (p = 0.02) were also observed, i.e., there was an increase of 5.1°.

The other objective regarding statistical analysis was to specify whether there existed a significant variation between age, sex, race, and type of immobilization *versus *chondrolysis. Statistical analysis was preformed by means of Fisher's accurate test, at 5% level [11]. Chondrolysis was present in 11.3% of the hips tested. There was no significant variation between age and chondrolysis (p = 1.00). Concerning gender analysis, statistically significant variations were observed (p = 0.031). In race analysis, there was also a statistically significant difference (p = 0.037). *No causal association between plaster cast and chondrolysis was observed *(p = 0.60). Regarding the symptomatology group and the slip degree *versus *chondrolysis, the p value was not statistically significant in either analysis, respectively p = 0.61 and p = 0.085.

## Discussion

The cause of articular cartilage necrosis after slipped capital femoral epiphysis still remains obscure [12]. Betz, Steel, Emper, Huss and Clancy found 13.5% of chondrolysis in their trials [7]. Ingram, Clarke, Clark and Marshall mentioned that the incidence of chondrolysis varies from 2% to 55% [6]. Jerre, in a series of 200 slipped femoral epiphyses treated mainly by closed reduction and plaster immobilization, found nine hips (4.5%) with articular cartilage necrosis [13]; in this study, chondrolysis affected 12 hips (11.3%): four presented a temporary form of chondrolysis (7.5%), with eight being permanent. Writings on this subject have shown a predominance of females over males [14,15]; in this series, chondrolysis was also predominant in females over males.

According to published works [2,14,16,17]; chondrolysis in non-white patients (16%- 66%) is more common than in white patients (2.5%). In this study, regarding articular cartilage necrosis, it was ascertained that non-white patients prevailed by a considerable number over the white patients. The manifestation and prevalence of chondrolysis as a complication in females and non-whites are some of the unclarified points in the study as of yet.

Regarding symptomatology, classification in previous studies assigns to chronic group patients the worst prognosis in relation to chondrolysis [6,7,17,18]. In this sample, the record of chondrolysis incidence in this type of group was in accordance with the literature.

Concerning the degree of epiphysis displacement in relation to the femoral neck, in chondrolysis, bad results are proportional to the severity of the slip degree [6,17,18]. In this study, seven patients classified as mild degree presented chondrolysis, five classified as moderate presented the complication, with none of the nine severe cases displaying it. This finding is contrary to the general condition.

Nevertheless, concerning chondrolysis, there was an inexplicable finding with one female patient who was treated for bilateral slipping by 1 1/2 spica cast. While her right hip was normal, the left one deteriorated to *chondrolysis*. Necrosis of articular cartilage is an entity that represents an auto-immune disease in genetically-susceptible individuals [19]. Still in relation to a chondrolysis complication, some authors affirm that excessive immobilization also favors articular cartilage necrosis [13,16,20]. It was observed, in this work, that five hips out of 12 were attacked by the disease when cast immobilization was used for over 12 weeks (apprehension curve).

Waldenström mentioned that the collum produces new vessels, which attempt to heal rupture continuity [20]. The period of immobilization (12 weeks) was observed as providing stability of the epiphysis to metaphysis, thus avoiding displacement continuity. Ponseti and Barta ascertained that growth plate obliteration process happens between 5 and 12 months, with a 9-month average after the beginning of the treatment with cast immobilization [16]. In this work, growth plate ossification time was 16.5 months.

Green found a 5% average progression of slipping after the cast had been discontinued (one of 18 hips; this patient's hip had been immobilized for only 8 weeks) [21]. Jerre found definite redisplacement in 20 (10%) hips in his series [13]. For prevention of additional slip of chronic SCFE groups, Betz, Steel, Emper, Huss and Clancy have shown effective treatment in 12 weeks, with a *spica *cast [7]. They reported one progression (8 weeks in a cast only) out of 37 hips. The range of time in which a redisplacement is possible is claimed by Waldenström to be approximately 1 year [22]. Wilson observed redisplacement occurring within 2 to 33 months (average, 11.8 months) from the start of the treatment [23]. In the present series, out of 106 hips, six (5.6%) were recorded with redisplacement (on average, 11 months after the cast had been removed), four following a traumatic episode.

King presented the use of bilateral short-leg cast immobilization as a form of treatment without chondrolysis [9]. In his work, 52 affected hips were recorded with satisfactory results. In the article, 33 short/long-leg casts in abduction and internal rotation were fixed with a stick; four chondrolysis were found, and, in 73 plaster *spica *casts, eight cases.

The disadvantages of immobilization in a *spica *cast include potential skin and pulmonary problems, ileus, and the difficulty in handling an obese child, in addition to problems involving education [7]. These disadvantages should be taken into consideration because of the risks of pinning by means of wires or screws, and the serious sequelae which include pin penetration, fracture, infection, pin breakage, growth disturbance, wound problems, subsequent slippage, difficulty in pin extraction during hardware removal, nail slipping into the joint, nail extruding, nails bending, avascular necrosis, as well as chondrolysis [7,14,15,24,25]. The global incidence of chondrolysis is 7% with all forms of treatment [26]. Chondrolysis can appear spontaneously after the slipping of the femoral epiphysis without any treatment, and may follow either a slight or a severe slip. It may occur after any type of treatment, whether conservative or operative [12].

*These results show why some methods are in favor, and others are in disfavor, in the clinic where these patients were treated and where as, in all hospitals the facilities and limitations must be evaluated by every surgeon (Clarence H. Heyman, M D) *[27].

## Conclusions

After analyzing the nonoperative treatment in slipped capital femoral epiphysis and chondrolysis, we concluded that the employment of the treatment revealed that the method was functional, efficient, valid, and reproducible; it can also be used as an alternative therapeutic procedure regarding to this specific disease.

This manuscript is faced with the fact that the orthopaedic surgeons employ and evaluate a little-adopted treatment technique by musculoskeletal studies in the treatment of SCFE. The success or failure of treatment intervention is determined based on the outcomes [28]. The presented work was evaluated and tested on its contents, methodology and clinical usefulness. Modern medicine is based on evidence, and outcomes have to have their importance proven. The instrument of quality employed (plaster cast method) was assessed not only by the surgeon, but also by the patient, through his descriptions. The patient was always given the option, upon the first appointment, to choose from the conservative or surgical treatment. The nonoperative management of SCFE was accepted by relatives. The interest demonstrated by the patients in method reliability has shown the possibility of analyzing the difference between the patients' reports, and those from the professionals and their studies, with the possibility of varied outcomes. Evaluation in modern medicine must be based on evidences of the result and on the functional radiographic measurements, in addition to being statistically analyzed and including the patients' reports. The present work showed an optional method for the treatment of slipped capital femoral epiphysis.

## Consent

Written informed consent was obtained from all patients and relevant parents/guardians for publication of this report and accompanying images. A copy of the written consent is available for review by the Editor-in-Chief of this journal.

## Competing interests

The author has not received any outside funding or grants in support for, or in preparation of his research. Neither did he, nor any member of his immediate family receive payments, benefits or agreements to provide the research for financial reasons.

## Authors' Information

The author certifies that he has no commercial associations (e.g. consultancies, stock holdings, equity interest, patent/licensing arrangements, etc) which might pose a conflict of interest in connection with the submitted article.
